# Ultrasound-Assisted Enzymatic Extraction of Mangiferin From Mango (*Mangifera indica* L.) Peels: Process Optimization and Antioxidant Activity Evaluation

**DOI:** 10.1155/jamc/3469361

**Published:** 2025-08-25

**Authors:** Kaiping Zhang, Yun Huang, Yanli Liu, Jiamin Ma, Qinjiabao Hu, Lingling He, Jiaoli Huang

**Affiliations:** ^1^College of Agriculture and Food Engineering, Baise University, Baise 533000, China; ^2^Guangxi Key Laboratory of Biology for Mango, Baise 533000, China; ^3^College of Food and Drug Engineering, Guangxi Vocational University of Agricultural, Nanning 533001, China

**Keywords:** bioactivity, extraction process, mangiferin, mango peels, ultrasound-assisted enzymatic method

## Abstract

Mango peel is one of the main byproducts in mango processing. In order to improve the utilization and added-value of mango peel, effects of the ultrasound-assisted enzymatic method on the extraction rate of mangiferin from mango peel were investigated by single-factor tests (enzyme addition amount, enzymolysis time, ethanol concentration, ultrasonic temperature, ultrasonic power, and ultrasonic time), and the extraction process was optimized by Box–Behnken response surface. The antioxidant activity and antidiabetic assay of mangiferin in vitro were studied. The results showed that enzymolysis time 44 min, enzyme addition amount 0.1%, ethanol concentration 70%, ultrasonic power 300 W, ultrasonic temperature 66°C, and ultrasonic time 45 min were the optimal extraction process parameters. Under these conditions, the extraction rate of mangiferin was 3.63% ± 0.04%, which was not significantly different from the model prediction value (3.71%), and it was 3.90 times and 1.78 times that of single cellulase and ultrasonic extraction, respectively. Within the test concentration range, the scavenging rate of mangiferin for DPPH·, ABTS^+^·, and O_2_^−^· increased with the increase of its mass concentration, and the EC_50_ values were 0.03829 mg/mL, 0.06032 mg/mL, and 0.04949 mg/mL, respectively, which were slightly higher than those of Vc. Meanwhile, mangiferin exhibited inhibitory activity effects on α-amylase and α-glucosidase, with IC_50_ values of 0.1952 and 0.08696 mg/mL, respectively. Results indicate that the mangiferin in mango peel had strong bioactivity. The study provides a theoretical reference for the efficient extraction of mangiferin from mango peel and provides a scientific basis for further realizing the high-value utilization of mango processing waste.

## 1. Introduction

Mango (*Mangifera indica* L.) belongs to the genus *Mangifera* of the Anacardiaceae family. It is rich in various vitamins and mineral elements, which are beneficial to human health. It is one of the most important tropical fruits in the world. According to statistics from the Food and Agriculture Organization (FAO) of the United Nations, global mango production in 2023 reached approximately 51.94 million tons. India ranked first worldwide with a mango output of 22.5 million tons [1], followed by China, which produced 3.9 million tons annually. Each year, several million tons of mango peel byproducts are generated from this production. In addition to being eaten fresh, mango can be processed into derivative products such as juice, concentrates, dried fruits, jam, fruit wine, and fruit vinegar [2–4]. Mango peels, which account for 15%–20% of the fruit weight, are one of the main byproducts in mango production and processing [5]. Several million tons of mango peel byproducts are produced each year, which are often discarded in landfills and are prone to microbial spoilage, posing a hazard to human health and the environment and the loss of valuable substances. The value-added of the byproducts of mango peel can be achieved by recycling high-value compounds, which is a sustainable way to solve the problem of the large amount of biological waste generated by the mango processing industry and its disposal.

Mango peel is a rich source of important nutrients and bioactive compounds. A number of natural bioactive substances have been reported in the mango peels including mangiferin, dietary fiber, pectin, flavonoids, and polyphenols [6–9]. These compounds due to their natural antioxidant capacity hold significant potential in the replacement of synthetic food additives and as a diabetes control agent. Mangiferin is the main bioactive component in mango peel, which has excellent anti-inflammatory [10], anticancer [11], and antioxidant functions [12]. It helps prevent various chronic diseases related to oxidative stress, such as diabetes [13], cardiovascular diseases [14], and neurodegenerative diseases [15]. As a plant-based antioxidant, mangiferin is superior to synthetic antioxidants due to its advantages such as low toxicity, good biocompatibility, and good biospecificity. In this context, the selection of the mangiferin extraction process and the optimization of the extraction process are crucial for maximizing the yield and bioactivity of mangiferin. However, due to the poor solubility and limited transmembrane permeability of mangiferin, as well as the numerous challenges faced in extracting mangiferin from mango peels, it is necessary to develop efficient, green, and environmentally friendly extraction technologies.

The traditional extraction methods of mangiferin include immersion extraction, alcohol extraction, and Soxhlet reflux extraction [16, 17]. However, traditional extraction methods suffer from several major drawbacks, including being time-consuming, requiring large volumes of solvent, exhibiting low extraction efficiency, and potentially altering the functional properties of mangiferin, thereby reducing its nutritional quality and causing secondary pollution. This has increased the interest of the scientific community in developing mild and environmentally friendly extraction techniques to replace traditional methods. To increase the extraction rate of mangiferin from mango peels, unconventional extraction techniques such as ultrasonic extraction [18], microwave extraction [19], and microwave-assisted enzymatic extraction were adopted to extract mangiferin from mango peels. Ultrasonic waves generate high-speed, intense cavitation, along with mechanical and thermal effects, which disrupt cell wall structures. This disruption facilitates rapid solvent penetration into the matrix, thereby accelerating mangiferin diffusion from the matrix to the solvent [20, 21]. In addition, cellulase enhances the speed and degree of cell wall structure destruction, further aiding mangiferin release. Ultrasound-assisted enzymatic extraction has been widely applied due to its advantages, including environmental friendliness, short extraction time, low solvent consumption, and high extraction efficiency [22]. However, there are no reports on the synergistic effect of enzymatic and ultrasonic treatment on the extraction of mangiferin from mango peel.

Using response surface methodology (RSM) based on the Box–Behnken design (BBD), a robust model can accurately fit the quadratic relationships between factors and response values, efficiently capture interaction effects, and predict optimal process parameters, thereby significantly improving the extraction rate of mangiferin [23]. Compared to traditional methods, this approach substantially reduces the number of experiments while rendering the results more scientific, reliable, and practical. It is particularly suitable for optimizing the extraction of natural products. Therefore, this research utilized an ultrasound-assisted enzymatic method to extract mangiferin from mango peel, and the extraction process was optimized to improve the extraction rate of mangiferin using the RSM with BBD. Meanwhile, the antioxidant activity and antidiabetic activity (in vitro) of mangiferin in mango peel were systematically investigated. The study aimed to explore novel approaches for efficient mangiferin extraction from mango peel and provide a theoretical foundation for the high-value utilization of mango processing byproducts.

## 2. Materials and Methods

### 2.1. Reagents and Solvents

Mangiferin standard (HPLC 98%) was purchased from Shanghai Shifeng Biological Technology Co., Ltd. (Shanghai, China); cellulase (10,000 U/g, BR) was purchased from Nanning Shanwan Biotechnology Co., Ltd. (Nanning, China); α-amylase enzyme (100 U/g, BR) was purchased from Sigma-Aldrich (St. Louis, MO, USA); α-glucosidase enzyme (50 U/mg, BR) and 4-nitrophenyl alpha-D-glucosaminide (PNPG) (98%) were purchased from Shanghai Shifeng Biotechnology Co., Ltd. (Shanghai, China); 1,1-diphenyl-2-picrylhydrazyl (DPPH·) was purchased from Hefei Qiansheng Biotechnology Co., Ltd. (Hefei, China); methanol and phosphoric acid (HPLC 98%) were purchased from Shanghai Macklin Biochemical Technology Co., Ltd. (Shanghai, China); Tris-HCl and ascorbic acid (Vc) were purchased from Hefei Beauty Biotechnology Co., Ltd. (Hefei, China); 2,2′-azinobis(3-ethylbenzothiazoline-6-sulfonic acid) (ABTS+·) was purchased from Beijing Solarbio Technology Co., Ltd. (Beijing, China); other reagents are analytically pure.

### 2.2. Experimental Materials

For this study, mangoes (*Mangifera indica* L.) of the Tainong No. 1 variety were selected, which were collected from Youjiang District, Baise City, China, in July 2024. Mango peels were collected from fresh and ripe mangoes with 14–17° Brix, with smooth peels and without disease and insect pests. The peels were then cleaned, adhesive pulp removed, cut into small pieces, and dried using a constant temperature blast drying box (DHG-9023A, Shenzhen Sanuo Instrument and Equipment factory, Shenzhen, China) at 60 ± 2°C until constant weight. The dried mango peel samples were crushed using a grinder (DFY-300, Wenzhou Dingli Medical Equipment Co., Ltd, Zhejiang, China) and then milled into powdered particles by using an electric mill (CHY-6001, Jinhua Mofei Home Appliance Co., Ltd, Zhejiang, China) and sieved using a No. 60 mesh. The mango peel powder samples were sealed and stored refrigerated at 4°C for subsequent research.

### 2.3. Ultrasound-Assisted Enzymatic Extraction of Mangiferin From Mango Peel

An ultrasonication technique assisted by enzymes was employed to extract mangiferin from mango peels [7]. 1.0 g of mango peel powder was weighed into a 150 mL conical flask, to which 40 mL of 0.2 mol/L acetic acid-sodium acetate buffer (pH 4.8) and 0.1% cellulase (based on the total amount of material and liquid) were added. The mixture underwent enzymolysis at 50°C and 120 r/min for 60 min in an oscillator (SHZ-82A, Changzhou Jinnan Instrument Manufacturing Co., Ltd, Jiangsu, China). Following enzymolysis, the mixture was immediately immersed in a 92°C–95°C water bath for 5 min. After cooling to room temperature, 40 mL of 70% ethanol was added and then kept in an ultrasound equipment (KQ5200DE, Kunshan Ultrasonic Instrument Co., Ltd, Jiangsu, China), which was subjected to ultrasonic extraction for 45 min at 55°C and 300 W. The extracted samples were centrifuged at 3500 r/min for 10 min. The supernatant was collected, and 2 mL of the supernatant was transferred to a volumetric flask and then diluted to 100 mL with 70% ethanol, and thus the test sample was formed. One milliliter of the test samples was analyzed according to the linear relationship between the mangiferin concentration and absorbance, and the extraction rate of mangiferin from mango peels was calculated using the following formula:

(1) $$\begin{array}{c} Y=\frac{c\times V\times n\times 10^{-6}}{m}\times 100\%, \end{array}$$

where *Y* is the extraction rate of mangiferin (%), *c* is the mass concentration of mangiferin (μg/mL), *V* is the volume (mL) of the sample solution, *n* is the dilution multiple, and *m* is the weight (*g*) of the mango peel powder.

### 2.4. Negative Control Test

Three samples of mangiferin standard, each weighing 1.0 g (accurate to 0.0001 g), were prepared for analysis. The test solution samples were prepared according to the extraction conditions in Section 2.3., and the absorbance was measured at 319 nm using an ultraviolet spectrophotometer (Shimadzu Instruments [Suzhou] Co., Ltd.), and the content of mangiferin was calculated. Meanwhile, 10 μg/mL of mangiferin solution treated with ultrasound-assisted enzymes and 6 μg/mL of mangiferin standard solution were taken, and their absorption spectra were investigated in 200.0–800.0 nm. The integrity of the mangiferin standard was validated based on these measurements.

### 2.5. Method Validation

#### 2.5.1. Linearity

Various concentrations of mangiferin standard solution (1, 2, 4, 6, 8, and 10 μg/mL) were accurately prepared, and the absorbance values were taken at 319 nm using an ultraviolet spectrophotometer. Standard curves were drawn with the absorbance corresponding to the mangiferin concentration and by utilizing linear regression analysis.

#### 2.5.2. Precision Test

Six samples with a concentration of 8 μg/mL were analyzed for absorbance at 319 nm to determine the mangiferin extraction rate and calculate the relative standard deviation (RSD) values.

#### 2.5.3. Repeatability Test

Following the method outlined by Zhu et al. [18], six samples of mango peel powder, each weighing 1.0 g (accurate to 0.0001 g), were prepared for analysis. The test solution samples were prepared according to the extraction conditions in Section 2.3, and the absorbance was measured at 319 nm. The mangiferin extraction rate and RSD values were then calculated based on these measurements.

#### 2.5.4. Stability Test

Following the method described by Zhu et al. [18], 1.0 g of mango peel powder was weighed to an accuracy of 0.0001 g. The test solution samples were prepared according to the extraction conditions in Section 2.3, and the absorbance of the mangiferin extract was measured at 319 nm, with an interval of 1 h, and the measurement was conducted continuously for 6 times. Subsequently, the mangiferin extraction rate and RSD values were calculated.

#### 2.5.5. Sample Recovery Test

Following the method described by Adin et al. [24], 1.0 g samples of mango peel powder were weighed accurately to 0.0001 g. A predetermined amount of mangiferin standard was added to each sample, and the test solution samples were prepared according to the extraction conditions in Section 2.3, and the absorbance was measured at 319 nm. The content and recovery of mangiferin were calculated using the following formula:

(2) $$\begin{array}{c} P=\frac{C_{2}-C_{1}}{M}\times 100\%, \end{array}$$

where *P* is the recovery (%), *C*_1_ is the raw mangiferin (mg/g), *C*_2_ is the mangiferin (mg/g), and *M* is the scalation (mg).

### 2.6. Single-Factor Experiments

Selecting appropriate experimental factors and working ranges facilitates enhanced mangiferin extraction rate. Through the preliminary experiment, the test conditions for ultrasonic-assisted enzymatic extraction of mangiferin were preliminarily determined as follows: enzymolysis time 60 min, enzyme addition amount 0.1%, ethanol concentration 70%, ultrasonic power 300 W, ultrasonic temperature 55°C, and ultrasonic time 45 min.

The factors and their levels were selected based on the results of our preliminary experiments (data not reported). The single-factor rotation method was used with the extraction rate of mangiferin as the evaluation index. The effects of enzymolysis time (20, 40, 60, 80, and 100 min), enzyme addition amount (0.06%, 0.08%, 0.1%, 0.12%, and 0.14%), ethanol concentration (60%, 65%, 70%, 75%, and 80%), ultrasonic power (180, 240, 300, 360, and 420 W), ultrasonic temperature (35°C, 45°C, 55°C, 65°C, and 75°C), and ultrasonic time (25, 35, 45, 55, and 65 min) on the extraction rate of mangiferin were investigated.

### 2.7. Response Surface Test

Based on the single-factor experiments, the extraction rate of mangiferin (Y) as the response value, the enzymolysis time (A), enzyme addition amount (B), ethanol concentration (C), and ultrasonic temperature (D) were selected as the independent variables. According to the Box–Behnken of central composite in the Design-Expert 10.0.3.1 software, a total of 29 experiments with 4 factors and 3 levels were designed to optimize the process parameters for the ultrasound-assisted enzymatic extraction of mangiferin. The factors and their corresponding levels are presented in Table 1.

### 2.8. UHPLC Analysis

Following the method described by Kaur et al. [12], with minor modifications, the Shimadzu LC-40x UHPLC system, equipped with a PDA detector, was used for the analysis. Working solutions of the mangiferin standard were prepared at 1.0–10.0 ppm concentrations, thoroughly mixed by an oscillator for 1 min, and then ultrasonicated in an ice bath for 30 min. After ultrasonic treatment, the mixture was centrifuged at 4000 r/min for 10 min, and the supernatant was collected and filtered through a 0.45 μm membrane before liquid chromatography analysis. The chromatographic column was equilibrated for 30 min prior to injection. Chromatographic separation was carried out using an Agilent Eclipse XDB-C18 column (250 × 4.6 mm, 5 μm particle size). The mangiferin extract obtained under the optimal extraction conditions was treated according to the above steps. The injection volume was set at 10 μL, and the mobile phase consisted of methanol (A) and 0.2% phosphoric acid aqueous solution (B), with a ratio of 20:80. The column temperature was set at 30°C, the wavelength was set at 319 nm, the flow rate was 1 mL/min, and the elution time was 30 min. Taking the peak area as the vertical coordinate and the injection amount of mangiferin standard as the horizontal coordinate, the regression equation of a standard mangiferin was *y* = 32114*x* − 18.153, with a correlation coefficient (*R*^2^) of 0.9997. These results indicate a strong correlation between the peak area and concentration of mangiferin. Meanwhile, the retention time and concentration of samples were compared with those of the mangiferin standard for quantification and characterization analysis.

### 2.9. Determination of Antioxidant Activity

For the determination of antioxidant activity, a 0.4 mg/mL mangiferin sample solution was prepared using an appropriate amount of mangiferin crude extract. The EC_50_ value represents the concentration of mangiferin required to achieve a 50% maximal free radical scavenging rate, measured in mg/mL. A lower EC_50_ value indicates a stronger antioxidant capacity of the mangiferin crude extract.

#### 2.9.1. Determination of the DPPH· Scavenging Rate

Following the method described by Khumpook et al. [25], with minor modifications, a specified quantity of mangiferin sample solution was measured. After content adjustment, mangiferin diluents were precisely prepared with mass concentrations of 0.02, 0.04, 0.06, 0.08, 0.1, 0.2, 0.3, and 0.4 mg/mL. Subsequently, mangiferin diluent (2 mL) and 0.1 mg/mL DPPH· solution (2 mL) were mixed in a test tube and shaken vigorously. The tubes were maintained at light-avoidance reaction for 30 min, and the absorbance was taken at 517 nm, denoted as *A*_1_. *A*_0_ was the absorbance value taken at 517 nm when the absolute ethanol was used to replace the mangiferin diluent. *A*_2_ was the absorbance value taken at 517 nm when the absolute ethanol was used to replace the DPPH· solution. Ascorbic acid (Vc) at identical mass concentrations served as a positive control. The DPPH· scavenging rate was calculated by using the following formula:

(3) $$\begin{array}{c} \frac{\text{DPPH}\cdot \text{scavenging rate}}{\%}=\left[1-\frac{A_{1}-A_{2}}{A_{0}}\right]\times 100. \end{array}$$

#### 2.9.2. Determination of the ABTS^+^· Scavenging Rate

The ABTS^+^· assay was performed according to the method of Uuh-Narváez et al. [9], with some modifications. 20 mL of 7 mmol/L ABTS^+^· solution and 20 mL of 4.9 mmol/L potassium persulfate solution were combined in a reagent bottle. The mixture was allowed to stand in the dark at 4°C for 14 h to obtain the ABTS^+^· stock solution. The stock solution was diluted with absolute ethanol to achieve an absorbance value of 0.7 ± 0.03 at a wavelength of 734 nm, resulting in the ABTS^+^· working solution. Subsequently, 1 mL of mangiferin dilutions with mass concentrations of 0.02, 0.04, 0.06, 0.08, 0.1, 0.2, 0.3, and 0.4 mg/mL were accurately measured. To each dilution, 5 mL of the ABTS^+^· working solution was added and thoroughly mixed. The reaction mixtures were allowed to react in the dark at room temperature for 6 min, and the absorbance was taken at 734 nm, denoted as *A*_1_, after the adjustment of the absorbance value to 0 with absolute ethanol. *A*_0_ was the absorbance value measured at 734 nm when the absolute ethanol was used to replace the mangiferin diluent. *A*_2_ was the absorbance value measured at 734 nm when the absolute ethanol was used to replace the ABTS^+^· working solution. Ascorbic acid (Vc) with the same mass concentration was used as a positive control, and the ABTS^+^· scavenging rate was calculated according to the following formula:

(4) $$\begin{array}{c} \frac{\text{ABT}S^{+}\cdot \text{ scavenging rate}}{\%}=\left[1-\frac{A_{1}-A_{2}}{A_{0}}\right]\times 100. \end{array}$$

#### 2.9.3. Determination of the O_2_^−^· Scavenging Rate

The O_2_^−^· scavenging rate was determined through the phenanthrene autoxidation method outlined by Yang et al. [26], with minor modifications, Precisely 1 mL of mangiferin diluents with mass concentrations of 0.02, 0.04, 0.06, 0.08, 0.1, 0.2, 0.3, and 0.4 mg/mL were measured and transferred into test tubes. Subsequently, 2.5 mL of Tris–HCl buffer solution (pH 8.2, 0.1 mol/L) and 0.5 mL of 25 mmol/L pyrogallic acid solution were added successively. The mixtures were homogenized using a vortex oscillator and allowed to stand at room temperature for 20 min. Then, immediately, 0.1 mL of concentrated hydrochloric acid was added to terminate the reaction. The spectrophotometer was calibrated to zero using a 10 mmol/L hydrochloric acid solution, and the absorbance was taken at 320 nm, denoted as *A*_1_. *A*_0_ represents the absorbance value when a hydrochloric acid solution was used in place of the mangiferin dilution. *A*_2_ represents the absorbance value when the hydrochloric acid solution was used in place of the pyrogallol solution. Ascorbic acid (Vc) at the same mass concentration served as the positive control. The scavenging rate of O_2_^−^· was calculated as

(5) $$\begin{array}{c} \frac{O_{2}^{-}\cdot \text{ scavenging rate}}{\%}=\left[1-\frac{A_{1}-A_{2}}{A_{0}}\right]\times 100. \end{array}$$

#### 2.9.4. Determination of the Total Reducing Capacity

The total reducing capacity assay was performed according to the method of Jiang et al. [27], with slight modifications. 2.5 mL of mangiferin dilutions with mass concentrations of 0.02, 0.04, 0.06, 0.08, 0.1, 0.2, 0.3, and 0.4 mg/mL were accurately measured and transferred into test tubes. To each test tube, 0.2 mol/L pH 6.6 phosphate buffer solution (2.5 mL) (PBS) and 1% K_3_[Fe(CN)_6_] solution pH (2.5 mL) were added successively and mixed thoroughly. The mixtures were incubated in a 50°C water bath for 30 min. Afterward, the mixtures were quickly cooled, and a 10% TCA solution (2.5 mL) was added. The reaction mixture was centrifuged at 3000 r/min for 10 min, collecting the supernatant of 5 mL into a separate test tube. Subsequently, 0.1% FeCl_3_ solution (2.5 mL) and distilled water (2.5 mL) were added to the test tube. The solutions were mixed well and allowed to stand for 10 min, and the absorbance was taken at 700 nm. Ascorbic acid (Vc) at the same mass concentration served as the positive control.

### 2.10. Antidiabetic Assay (In Vitro)

For the determination of in vitro antidiabetic activity, first, the mangiferin dilutions with mass concentrations of 0.02, 0.04, 0.06, 0.08, 0.1, 0.2, 0.3, and 0.4 mg/mL were accurately measured with 0.1 mol/L pH 6.6 PBS, and the inhibitory activity of α-amylase enzyme and α-glucosidase was determined to check the in vitro antidiabetic assay. The IC_50_ value represents the concentration of mangiferin required to reduce α-amylase enzyme or α-glucosidase activity by 50%, measured in mg/mL. A lower IC_50_ value indicates a stronger inhibition capacity of the mangiferin crude extract.

#### 2.10.1. Determination of the Inhibitory Ability of Mangiferin Extracts on α-Amylase Enzyme Activity

Following the method described by Kaur et al. [12], with minor modifications, 1.0 mL of mangiferin dilutions and 1.0 mL of 5 U/mL α-amylase enzyme solution were accurately measured and transferred into test tubes and incubated at 37°C for 20 min. 1.0 mL of 0.5% starch solution was added to the test tubes and incubated at 37°C for 10 min. After preincubation, 5.0 mL of 3,5-dinitrosalicylic acid (DNS) color reagent was added and incubated in a boiling water bath for 5 min, cooled to room temperature, and the absorbance was taken at 540 nm, denoted as *A*_1_. *A*_0_ was the control group (PBS in place of samples); *A*_2_ was the blank group (PBS in place of the enzyme solution). Acarbose at the same mass concentration served as the positive control. The inhibition rate of α-amylase enzyme activity was calculated as follows:

(6) $$\begin{array}{c} \frac{\alpha -\text{amylase inhibition rate}}{\%}=\left[1-\frac{A_{1}-A_{2}}{A_{0}}\right]\times 100. \end{array}$$

#### 2.10.2. Determination of the Inhibitory Ability of Mangiferin Extracts on α-Glucosidase Enzyme Activity

Following the method described by Medina-Saavedra et al. [28], with minor modifications, 1.0 mL of mangiferin dilutions with different concentrations and 1.0 mL of 2.5 U/mL α-glucosidase enzyme solution were accurately measured and transferred into test tubes and incubated at 37°C for 15 min. After preincubation, 1.0 mL of 2.5 mmol/L PNPG solution was added to the test tubes and incubated at 37°C for 10 min. Then, the reaction was stopped with 4.0 mL of 1.5 mol/L Na_2_CO_3_ solution, and the absorbance was taken at 405 nm, denoted as *A*_1_. *A*_0_ was the control group (PBS replaced with samples); *A*_2_ was the blank group (PBS replaced with enzyme solution). Acarbose at the same mass concentration served as the positive control. The inhibition rate of a-glucosidase enzyme activity was calculated as follows:

(7) $$\begin{array}{c} \frac{\alpha -\text{glucosidase inhibition rate}}{\%}=\left[1-\frac{A_{1}-A_{2}}{A_{0}}\right]\times 100. \end{array}$$

### 2.11. Data Analysis

All data were organized using Excel, with each test point repeated three times. All results were expressed as mean ± standard deviation. Response surface design and data analysis were carried out using the software Design-Expert 10.0.3.1, and one-way analysis of variance (ANOVA) was carried out using the software IBM SPSS Statistics 26.0. The differences between samples were compared using the least significant difference (LSD) test at the probability level of *p* < 0.05. The software Origin 2018 was used for drawing graphs.

## 3. Results and Discussion

### 3.1. Negative Control Test

**Figure 1:**
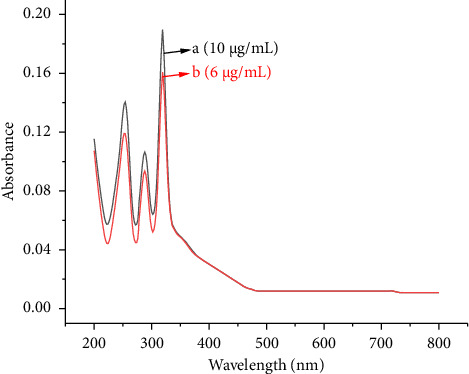
UV absorption spectrum of mangiferin standards after ultrasonication-assisted enzymatic treatment (a) and mangiferin standard (b).

Mangiferin standard samples were treated by ultrasound-assisted enzymatic technology. The content of mangiferin after processing is shown in Table 2, and the content of mangiferin was 0.99 ± 0.01 g, with the RSD being 0.8%. The UV absorption spectrum of mangiferin standards after ultrasound-assisted enzymatic treatment (a) and mangiferin standard (b) is shown in Figure 1. The absorption spectrum of two mangiferin standard solutions with different concentrations has the same peak shape, showing three absorption peaks in the range of 200.0–800.0 nm, which are at 254, 288 and 319 nm, respectively. Among them, the maximum absorption wavelength of mangiferin was 319 nm, which was consistent with the result (λmax = 319 nm) that had been reported by Huang et al. [29]. The results showed that the synergistic effect of cellulase and ultrasound had no effect on the degradation of mangiferin, and the original structural integrity of mangiferin was maintained, indicating that mangiferin had good stability.

### 3.2. Method Validation

#### 3.2.1. Linear Correlations

The absorbance values of mangiferin reference solutions with different mass concentrations were measured, and the mangiferin calibration curve was plotted. A regression equation (*y* = 0.0514*x* − 0.0026) (0–10 μg/mL) was fitted, and *R*^2^ = 0.9998. The results indicate that there is a good linear relationship between the mass concentration of mangiferin and absorbance, and this linear equation can be used to calculate the mangiferin content in the samples.

#### 3.2.2. Precision Test

The results of the precision test for ultrasonic-assisted enzymatic extraction of mangiferin are presented in Table 3. The data in Table 3 reveal an RSD of 0.50%, which falls below the 5% threshold. This value satisfies the precision standard, demonstrating that the instrument exhibits good precision. Consequently, ultraviolet spectrophotometry is deemed suitable for determining the extraction rate of mangiferin.

#### 3.2.3. Repeatability Test

The results of the repeatability test for ultrasonic-assisted enzymatic extraction of mangiferin are shown in Table 4. The data illustrate that the average extraction rate of mangiferin across six mango peel powder samples is 3.50 ± 0.02%, with an RSD of 0.55%. The values indicate that the experimental method demonstrates good repeatability.

#### 3.2.4. Stability Test

Table 5 presents the stability test results for mangiferin extracted via the ultrasonic-assisted enzymatic method. The data in Table 5 demonstrate an RSD of 0.24%, indicating that the mangiferin test solution exhibits good stability over a 5-h period.

#### 3.2.5. Recovery Test

Table 6 illustrates that the spiked recovery rate ranges from 96.19% to 101.61%, falling within the acceptance standard of 100 ± 15%. The average spiked recovery rate is 99.52 ± 1.97%, with an RSD of 1.98%. These findings align closely with previous studies on mangiferin content in mango leaves by Adin et al. [24], which reported recovery rates of 99.38%–99.92%, and in mango kernels by Zhu et al. [18], with rates ranging from 98.84% to 100.40%. These results demonstrate that this analytical method exhibits high recovery rates, good accuracy, and reliability, indicating that the measured values closely approximate the true values.

### 3.3. Single-Factor Experiments for Mangiferin Extraction

#### 3.3.1. Effect of Different Enzymolysis Times on the Extraction Rate of Mangiferin

**Figure 2:**
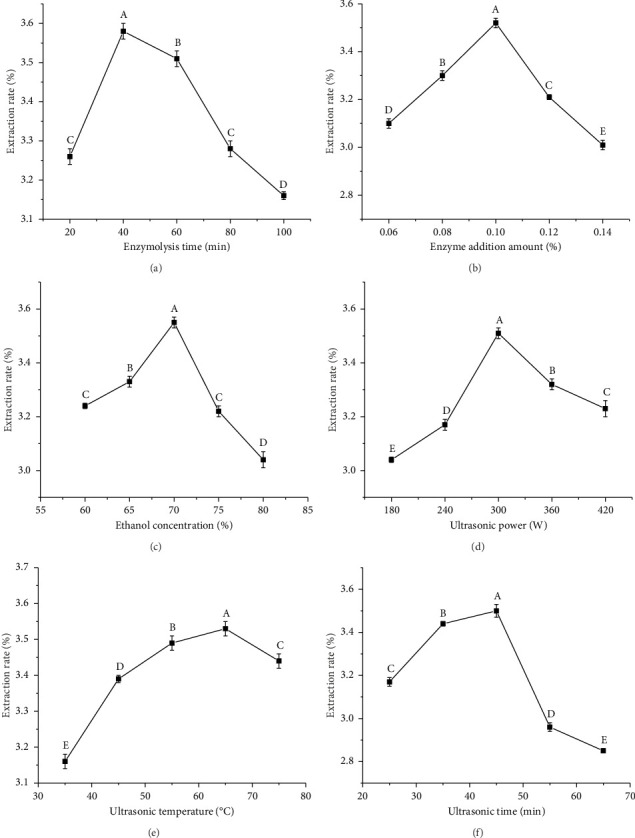
Effect of enzymolysis time (a), enzyme addition amount (b), ethanol concentration (c), ultrasound power (d), ultrasonic temperature (e), and ultrasound time (f) on the rate of mangiferin extraction from *Mangifera indica* L. peels. Note: Different letters indicate significant differences (*n* = 3, *p* < 0.05).

Figure 2(a) illustrates the impact of enzymolysis duration on the mangiferin extraction rate. The data indicate that the extraction rate initially increases and subsequently decreases gradually as the enzymolysis time extends. The highest extraction rate of mangiferin, 3.55% ± 0.02%, is achieved at an enzymolysis time of 40 min. This phenomenon can be explained by the incomplete hydrolysis of the cell wall by cellulase during short enzymolysis periods, which impedes mangiferin release from the cells, resulting in a low extraction rate. As the enzymolysis time increases, the cell wall undergoes complete lysis, leading to a looser cell structure and enhanced mangiferin dissolution, thereby increasing the extraction rate. However, excessively prolonged enzymolysis may result in the dissolution of more impurities, potentially inhibiting cellulase enzyme activity and consequently reducing the mangiferin extraction rate. This result is consistent with the report of Lu et al. [6] on the extraction of mangiferin from mango peel residue using microwave-assisted cellulase. According to these observations, three enzymolysis durations, 20, 40, and 60 min, are selected for the response surface optimization test.

#### 3.3.2. Effect of Different Enzyme Addition Amounts on the Extraction Rate of Mangiferin

The influence of enzyme addition on the mangiferin extraction rate is illustrated in Figure 2(b). As the cellulase concentration increases from 0.06% to 0.1%, the mangiferin extraction rate increases significantly (*p* < 0.05). The extraction rate reaches its peak of 3.52% ± 0.02% at a cellulase concentration of 0.1%. Further increases in cellulase concentration lead to a significant decrease in the mangiferin extraction rate. This phenomenon can be attributed to the relationship between enzyme concentration and substrate interaction. At low cellulase concentrations, the limited contact area between enzyme and substrate results in weak enzymolysis, insufficient cell wall degradation, and consequently, poor solvent penetration, leading to low extraction rates. As the enzyme concentration increases, the expanded contact area between enzyme and substrate accelerates cell wall dissolution and enhances cell membrane permeability [30], facilitating the release of mangiferin from plant cells and increasing yield. However, excessive cellulase concentrations may excessively hydrolyze cell wall polysaccharides, releasing more impurities and thereby reducing mangiferin extraction efficiency. The optimal cellulase dosage selected in this study was 0.1%. This finding is inconsistent with the previous studies optimizing antioxidant extraction from fruit byproducts; other researchers used cellulase concentrations ranging from 0.16% to 2% [27, 31], These may be related to differences in plant substrates and cellulase characteristics. Furthermore, there are limited reports on the enzymatic extraction of mangiferin from mango peels. Multiple comparisons via the LSD method reveal that the mangiferin extraction rate at 0.1% cellulase concentration differs significantly from those at other enzyme concentrations (*p* < 0.05). Based on these findings, cellulase concentrations of 0.08%, 0.1%, and 0.12% are selected for further optimization through RSM.

#### 3.3.3. Effects of Different Ethanol Concentrations on the Extraction Rate of Mangiferin

Preliminary screening experiments (not shown) were carried out using different polar solvents such as methanol, anhydrous ethanol, 50% ethanol solution, ethyl acetate, and acetone for mangiferin extraction, and it was found that the content of mangiferin in 50% ethanol was significantly higher than that of other extractants, which was consistent with the results of Kaur et al. [12]. Ethanol has been found to have the highest affinity for phenolics, which is inexpensive, nontoxic and environmentally benign, making it a preferred solvent for extracting phenolic compounds from various plants, and plays a crucial role in mangiferin extraction.  Figure 2(c) illustrates that within the ethanol concentration range of 60%–70%, the extraction rate of mangiferin increases with higher ethanol concentrations. This trend is attributable to ethanol's ability to induce polarity in mangiferin. Following the principle of “like dissolves like,” mangiferin's solubility in ethanol increases. In addition, higher ethanol concentrations cause greater damage to cell membranes, enhancing mangiferin's diffusion rate and consequently increasing its extraction yield. However, when ethanol concentration exceeds 70%, the mangiferin extraction rate gradually decreases. This decline possibly occurs because less polar alcohol-soluble substances in mango peel compete with mangiferin for dissolution. Furthermore, mangiferin is soluble in hot dilute ethanol, and high-concentration ethanol reduces its solubility [32]. Multiple comparison analysis based on the LSD method reveals that at 70% ethanol concentration, the mangiferin extraction rate reaches its maximum value of 3.55% ± 0.02%, which is significantly higher than those under ethanol concentrations (*p* < 0.05). Consequently, three levels of ethanol concentration, 65%, 70%, and 75%, are selected for the response surface optimization test.

#### 3.3.4. Effects of Different Ultrasonic Power on the Extraction Rate of Mangiferin

Figure 2(d) illustrates the influence of ultrasonic power on the mangiferin extraction rate. Within the range of 180–300 W, the extraction rate of mangiferin increases significantly with rising ultrasonic power (*p* < 0.05). This phenomenon occurs because ultrasonic wave propagation in the solvent generates compression and rarefaction cycles, creating numerous cavitation bubbles. These bubbles, existing for less than a single cycle, collapse violently, causing cavity implosion. This process results in material pore formation, enhanced absorption, cell expansion, softness, and looseness, thereby increasing solvent penetration into the matrix and facilitating the release of cell contents into the solvent [33]. In addition, cavitation produces microjets, shock waves, and turbulence, which damage plant cell walls, enhance solvent diffusion into the matrix, increase mangiferin solubility, and consequently increase the mass transfer rate of mangiferin into the solvent and the extraction rate [34]. However, when ultrasonic power exceeds 300 W, the thermal effect generated by high ultrasonic power may damage the mangiferin structure. Furthermore, the strong mechanical effect produced by high ultrasonic power accelerates the extract flow, reducing the ultrasonic wave residence time. Concurrently, the enhanced cavitation effect generates numerous useless cavitation bubbles, increasing ultrasonic wave scattering attenuation and resulting in decreased mangiferin extraction rate. Compared to other single factors, ultrasonic power has a relatively minor influence on the mangiferin extraction rate. This result is consistent with the results of Yang et al. [26] studying flavonoids from *Rosa sterilis* pomace and Xue et al. [35] studying anthocyanins from raspberry wine residues. Therefore, in subsequent response surface tests, the ultrasonic power is fixed at 300 W.

#### 3.3.5. Effects of Different Ultrasonic Temperatures on the Extraction Rate of Mangiferin

Temperature significantly influences the extraction of bioactive compounds [7]. Elevating the extraction temperature enhances the solubility and diffusion coefficient of mangiferin, facilitating its extraction.  Figure 2(e) illustrates that within the ultrasonic temperature range of 35°C–65°C, the mangiferin extraction rate increases rapidly with rising temperature. At 65°C, the mangiferin extraction rate peaks at 3.53% ± 0.02%. Further temperature increases lead to a decrease in the extraction rate. This phenomenon possibly occurs because at lower temperatures, the solvent's vapor pressure is low, resulting in fewer cavitation bubbles [34]. As the temperature rises, the solvent's vapor pressure increases, generating more cavitation bubbles and enhancing acoustic cavitation. This process ruptures plant cells and accelerates the mass transfer of mangiferin. In addition, higher temperatures improve solvent diffusion rates and mangiferin solubility, expediting mangiferin penetration from cells and yielding higher extraction rates. However, when temperatures exceed 65°C, the solvent's surface tension and viscosity decrease, weakening acoustic cavitation and consequently reducing extraction rates. Moreover, high temperatures can oxidize mangiferin's hydroxyl group, compromising its structure and further diminishing extraction rates. Similar results were obtained by Sharif et al. [22], who extracted mango polyphenols from mango peel waste by the ultrasound-assisted protease method using different temperature treatments. Multiple comparisons using the LSD method reveal that the mangiferin extraction rate at 65°C is significantly higher than at other ultrasonic temperatures (*p* < 0.05). Based on these findings, three ultrasonic temperature levels, 55, 65, and 75°C, are selected for the response surface optimization test.

#### 3.3.6. Effects of Different Ultrasonic Times on the Extraction Rate of Mangiferin

The contact time between the extraction solvent and the plant matrix significantly influences the gradual release of metabolites and the efficiency of the extraction process.  Figure 2(f) shows that the extraction rate of mangiferin showed an increasing trend and then decreased with the increase of ultrasonic temperature, which was similar to the results of Zhu et al. [18]. When the ultrasonic time increases from 25 to 45 min, the extraction rate of mangiferin rises from 3.17% to 3.51%. This increase is attributed to the thermal effect generated by the ultrasound, which causes rapid vaporization of water molecules in plant cells. The resulting pressure creates pores in the plant cell membrane and cell wall [7]. In addition, the mechanical shear force produced by ultrasound disrupts the cell wall structure, facilitating rapid solvent penetration into the cells and accelerating the mass transfer of cellular contents to the solvent [35]. However, when the ultrasonic duration exceeds 45 min, the extraction rate of mangiferin decreases. This decline is attributable to the oxidation and decomposition of mangiferin caused by prolonged ultrasonic treatment, as well as increased solvent viscosity and mass transfer resistance, which impede mangiferin extraction. Multiple comparisons via the LSD method reveal that ultrasonic durations exceeding 45 min yield significantly higher mangiferin extraction rates than other durations (*p* < 0.05). Notably, ultrasonic duration has a relatively minor impact on the mangiferin extraction rate compared to other single factors. Consequently, the ultrasonic duration is set at 45 min for subsequent response surface tests.

### 3.4. Response Surface Optimization Test Results

The experimental data of 29 runs conducted using the RSM Box–Behnken central composite design are presented in Table 7.

#### 3.4.1. Establishment of the Response Surface Regression Model Equation and Variance Analysis

Following multiple regression analysis of the experimental data in Table 7 using Design-Expert 10.0.3.1 software, the regression equation for mangiferin extraction rate (Y) in relation to enzymolysis time (A), enzyme addition amount (B), ethanol concentration (C), and ultrasonic temperature (D) is expressed as follows:

(8) $$\begin{array}{c} Y=3.69+0.085A-0.021B+0.099C+0.058D+0.060\text{AB}-0.037\text{AC}-0.018\text{AD}\\ -0.022\text{BC}-0.015\text{BD}-0.12\text{CD}-0.20A^{2}-0.20B^{2}-0.30C^{2}-0.22D^{2}. \end{array}$$

The ANOVA results for the regression model are detailed in Table 8. The table demonstrates that the regression equation model for the mangiferin extraction rate response value is highly significant (*F* = 32.88, *p* < 0.0001), while the lack-of-fit term is not significant (*F* = 0.61, *p* > 0.05). This suggests that the model fits well with the experimental factors, and the difference between the experimental value and the calculated value of the fitting equation is not significant, confirming the model's reliability. Consequently, this model and equation can be utilized to analyze and predict the mangiferin extraction rate in mango peel under various process conditions. The model's determination coefficient *R*^2^ is 0.9705, with values closer to 1 indicating a better model fit. The adjusted (*R*_Adj_^2^) and the predicted (*R*_Pred_^2^) determination coefficients were 0.9410 and 0.8789, and *R*_Pred_^2^-*R*_Pred_^2^ = 0.0621 < 0.2. Generally, higher and closer values of *R*_Adj_^2^ and *R*_Pred_^2^ indicate a stronger correlation between the actual observed value and the model-predicted value [36]. The coefficient of variation is 1.58%, indicating good model reproducibility and high accuracy and reliability of the entire experiment. The model's signal-to-noise ratio (AdP) is 21.101 > 4.0, suggesting a strong response signal and the ability to analyze and predict experimental results of the true value [37]. Significance analysis of the regression coefficients reveals that the First-order terms A and C, the Interaction term CD, and all second-order terms have an extremely significant influence on the mangiferin extraction rate (*p* < 0.001). The First-order term D has a highly significant influence (*p* < 0.001), and the Interaction term AB has a significant influence (*p* < 0.05). According to the magnitude of the F value, the influence of the four factors on the mangiferin extraction rate follows the order: C > A > D > B, corresponding to ethanol concentration > enzymolysis time > ultrasonic temperature > enzyme addition amount.

#### 3.4.2. Response Surface Interaction Analysis

**Figure 3:**
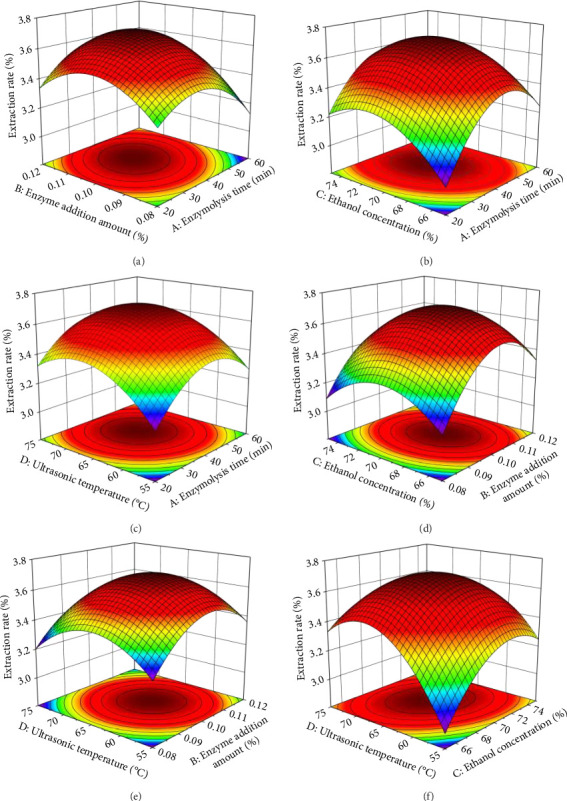
Response surface plots showing the effects of interactions between (a) enzymolysis time and enzyme addition amount, (b) enzymolysis time and ethanol concentration, (c) enzymolysis time and ultrasonic temperature, (d) enzyme addition amount and ethanol concentration, (e) enzyme addition amount and ultrasonic temperature, and (f) ethanol concentration and ultrasonic temperature on the extraction rate of mangiferin (*n* = 3).

According to the response surface experiment results, a three-dimensional response surface graph was generated using Design-Expert 10.0.3.1 software (Figure 3). The graph analyzes the interaction effects of four factors, enzymolysis time, enzyme addition amount, ethanol concentration, and ultrasonic temperature, on the mangiferin extraction rate. The steepness of the surface indicates the significance of the interaction; steeper surfaces suggest more significant interactions.  Figure 3(a) reveals a relatively steep slope for the surface of enzymolysis time and enzyme addition amount, indicating a significant interaction between these factors on the mangiferin extraction rate. The steeper slope for the enzymolysis time suggests that it has a greater influence than the enzyme addition amount.  Figure 3(f) displays the steepest slope for the surface of ethanol concentration and ultrasonic temperature, demonstrating an extremely significant interaction between these factors. The steeper slope for ethanol concentration indicates its greater influence compared to ultrasonic temperature. The response surfaces for other factor pairs, enzymolysis time and ethanol concentration, enzymolysis time and ultrasonic temperature, enzyme addition amount and ethanol concentration, and enzyme addition amount and ultrasonic temperature, are relatively gentle with near-circular contour lines. This suggests that the interactions between these pairs have less significant effects on the mangiferin extraction rate, consistent with the significance analysis results of the response surface regression model. According to Table 8, the influence of each interaction term on the mangiferin extraction rate in mango peel follows the order: CD > AB > AC > BC > AD > BD.

#### 3.4.3. Verification Test

Analysis using Design-Expert 10.0.3.1 software reveals that under the extraction conditions of 43.91 min of enzymolysis, 0.10% enzyme addition, 70.69% ethanol concentration, and 65.90°C ultrasonic temperature, the maximum theoretical mangiferin extraction rate reaches 3.71%. Although this value is lower than the rates observed in the first group (3.76%) and the 20th group (3.72%) (Table 7), the experimental conditions for these groups were repeated five times, yielding an average value of 3.69%, which is lower than the model prediction. Thus, the process conditions of the prediction value are adopted as the optimal extraction conditions. To verify the validity and sufficiency of the prediction model while considering operational feasibility and convenience, the optimal conditions for mangiferin extraction are adjusted as follows: 44 min enzymolysis duration, 0.10% enzyme addition, 70% ethanol concentration, and 66°C ultrasonic temperature. Three replicate experiments conducted under these modified extraction conditions yield a mangiferin extraction rate of 3.63% ± 0.04%. The relative error between the measured values and the theoretical values is relatively small, demonstrating high congruence with the model prediction and indicating good model fit and reliable experimental results. Under identical extraction conditions, mangiferin from mango peel extracted by the single cellulase method and the ultrasonic method alone yield extraction rates of 0.93% ± 0.02% and 2.04% ± 0.03%, respectively, suggesting that the ultrasonic-assisted cellulase method enhances mangiferin extraction efficiency from mango peel. This method has the advantages of simple operation, short extraction time, easy recovery of the extractant, no secondary pollution to the environment, and easy amplification. It can provide a scientific basis for the industrial production of mangiferin and help enterprises to achieve win–win economic and environmental benefits.

Huang et al. [29] obtained 4.622 mg/g of mangiferin from Keitt mango peels using microwave-assisted extraction. Lu et al. [6] achieved 6.229 mg/g of mangiferin from mango pomace using microwave-assisted cellulase extraction. Lerma-Torres et al. [16] obtained 1.45 g/100 g of mangiferin from mango leaves using ultrasonic treatment, while Morales et al. [21] achieved 314.34 mg/100 g extraction yield of mangiferin from ripe Criollo mango peels using ultrasound-assisted extraction. This study achieved a 3.63% extraction yield of mangiferin from mango peels through ultrasound-assisted cellulase extraction, indicating that this method significantly enhances mangiferin extraction efficiency and is also supposed to be an economical, rapid, and green extraction technology. This may not only serve as a sustainable development strategy for the food industry but also increase economic benefits by developing functional foods from extracted mangiferin. However, factors such as different producing areas, varieties, ripeness of mangoes, and extraction methods have a significant impact on the extraction rate of mangiferin, which requires further research.

#### 3.4.4. Analysis of the UHPLC-PDA Chromatogram of Mangiferin

**Figure 4:**
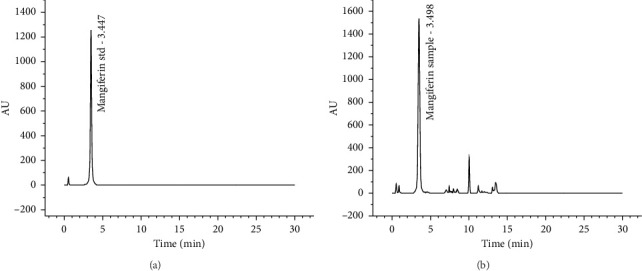
UHPLC-PDA chromatograms of mangiferin standard at 1.0 ppm (a) and mangiferin extracted from mango peel by ultrasonic-assisted enzymatic extraction at 2.0 ppm (b).

The UHPLC-PDA chromatograms of the mangiferin standard and mangiferin extracts are shown in Figure 4. The main chromatographic peak of mangiferin extracted from mango peel was retained at 3.498 min, while the standard mangiferin peak was detected at 3.447 min. Both peaks showed comparable retention times and maintained good separation from adjacent peaks, verifying the presence of mangiferin in mango peel, and the purity of mangiferin was 74.13%. The extraction rate of mangiferin was 3.63% ± 0.04%, lower than the 3.70% ± 0.03% measured by UV spectrophotometry. This discrepancy may result from other components with similar UV absorption properties (flavonoids or phenolic acids) coexisting in mango peel. These compounds may absorb light alongside mangiferin, leading to higher measured values.

### 3.5. Analysis of the Antioxidant Activity of Mangiferin

#### 3.5.1. Scavenging Effect of Mangiferin From Mango Peel and Vc on DPPH·

**Figure 5:**
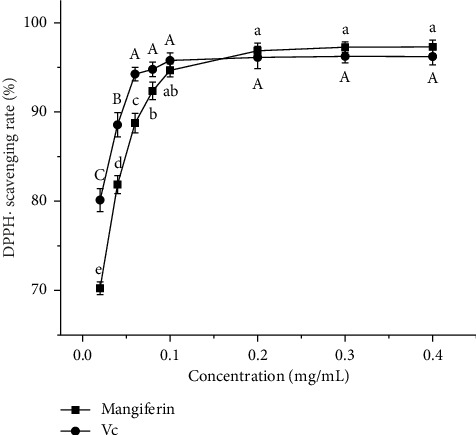
Scavenging effect of mangiferin from mango peel and Vc on DPPH·. Note: Different uppercase letters indicate significant differences in antioxidant activity between different mass concentrations of Vc (*p* < 0.05); different lowercase letters indicate significant differences in antioxidant activity between different mass concentrations of mangiferin (*p* < 0.05, the same below).

As depicted in Figure 5, the scavenging rates of mangiferin from mango peel and Vc on DPPH· increase proportionally with the rise in mass concentration of mangiferin and Vc. Under the Vc mass concentration range of 0.02–0.06 mg/mL, the scavenging rate of Vc on DPPH· increases significantly (*p* < 0.05) with the increase in Vc. When the Vc mass concentration exceeds 0.06 mg/mL, the scavenging rate stabilizes, remaining between 94.78% and 96.23%, with no significant difference in DPPH· scavenging rate among varying mass concentrations (*p* > 0.05). The EC_50_ of Vc on DPPH· scavenging rate is 0.03738 mg/mL. For mangiferin, within the mass concentration range of 0.02–0.08 mg/mL, the scavenging rate on DPPH· increases significantly (*p* < 0.05) with the increase in mangiferin. At concentrations above 0.08 mg/mL, the scavenging rate ranges from 92.36% to 97.29%, with no significant difference among varying mass concentrations (*p* > 0.05). In the range of 0.02–0.1 mg/mL, the scavenging rate of mangiferin on DPPH· is lower than that of Vc but higher than that of mangiferin extracted from mango leaves by Adin et al. [24] at the same concentration. However, in the 0.2–0.4 mg/mL range, the mangiferin extract's scavenging rate slightly surpasses that of Vc. This observation may be due to the presence of other antioxidant active substances in the mango peel extract, such as gallic acid, quercetin, anthocyanins, and carotenoids. The EC_50_ of the mangiferin extract's scavenging rate on DPPH· is 0.03829 mg/mL, lower than the EC_50_ reported by Khumpook et al. [25] (EC_50_ = 0.129 mg/mL) but slightly higher than that of Vc. This indicates that the mangiferin extract from mango peel exhibits potent antioxidant activity. These findings align with the trends observed in mangiferin's DPPH· scavenging activity reported by Ma et al. [38].

#### 3.5.2. Scavenging Effect of Mangiferin From Mango Peel and Vc on ABTS^+^·

**Figure 6:**
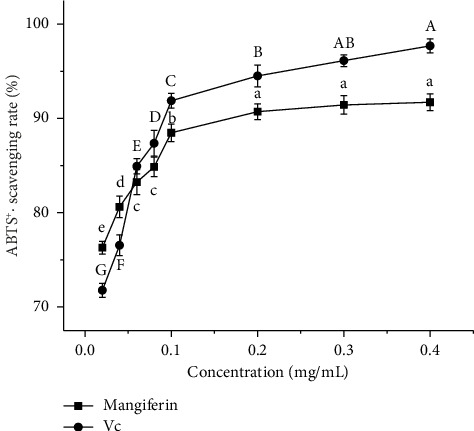
Scavenging effect of mangiferin from mango peel and Vc on ABTS^+^·. Different letters (A–G) and (a–e) in the same line indicate significant differences (*n* = 3, *p* < 0.05).

Figure 6 illustrates that the scavenging rates of ABTS^+^· by mangiferin extract and Vc increase progressively with higher mass concentrations of mangiferin and Vc. In the mass concentration range of 0.02–0.04 mg/mL, the mangiferin extract exhibits a higher ABTS^+^· scavenging rate than Vc. However, in the range of 0.06–0.4 mg/mL, Vc demonstrated superior scavenging activity. At 0.4 mg/mL, the mangiferin extract achieves a 91.72% scavenging rate, reaching 93.87% of Vc's scavenging rate (97.71%). The EC_50_ values for ABTS^+^· scavenging are 0.05845 mg/mL for Vc and 0.06032 mg/mL for mangiferin extract, indicating comparable efficacy. These values are lower than the EC_50_ (0.275 mg/mL) reported by Khumpook et al. [25] for mangiferin's ABTS^+^· scavenging activity. This discrepancy may be due to variations in antioxidant activities among different mango species and qualities from diverse geographical origins.

#### 3.5.3. Scavenging Effect of Mangiferin From Mango Peel and Vc on O_2_^−^·

**Figure 7:**
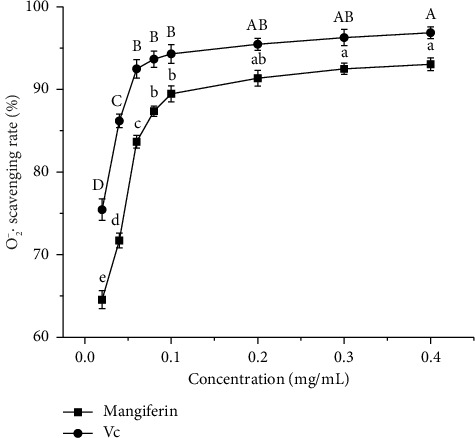
Scavenging effect of mangiferin from mango peel and Vc on O_2_^−^·. Different letters (A–D) and (a–e) in the same line indicate significant differences (*n* = 3, *p* < 0.05).

As illustrated in Figure 7, the scavenging rate of O_2_^−^· increases gradually with higher mass concentrations of mangiferin extract and Vc. At equivalent mass concentrations, the scavenging rate of O_2_^−^· by mangiferin extract is lower than that of Vc. For Vc concentrations between 0.02 and 0.06 mg/mL, the O_2_^−^· scavenging rate increases significantly (*p* < 0.05) from 75.44% to 92.49%. At concentrations above 0.06 mg/mL, the DPPH· scavenging rate by Vc stabilizes between 93.66% and 96.85%, with an EC_50_ of 0.03169 mg/mL for O_2_^−^· scavenging. For mangiferin concentrations between 0.02 and 0.08 mg/mL, the O_2_^−^· scavenging rate increases significantly (*p* < 0.05) from 64.55% to 87.37%. Beyond this range, the scavenging rate increases more slowly. At 0.4 mg/mL, the O_2_^−^· scavenging rate of mangiferin extract reaches a maximum of 93.04%, which is 96.06% of Vc's scavenging rate (98.65%) at the same concentration. The EC_50_ for O_2_^−^· scavenging by mangiferin extract is 0.04949 mg/mL, higher than that of Vc but substantially lower than 10 mg/mL. This indicates that mangiferin extract from mango peel possesses strong O_2_^−^· scavenging capabilities.

#### 3.5.4. Total Reducing Abilities of Mangiferin From Mango Peel and Vc

**Figure 8:**
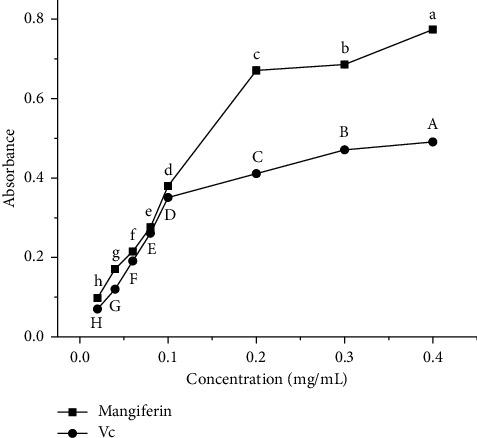
Reducing power of mangiferin from mango peel and Vc. Different letters (A–H) and (a–h) in the same line indicate significant differences (*n* = 3, *p* < 0.05).

Figure 8 demonstrates that within the mass concentration range of 0.02–0.4 mg/mL, the total reducing abilities of both mangiferin extract and Vc exhibit a significant upward trend as the mass concentration increases (*p* < 0.05). Throughout the analyzed range, the mangiferin extract consistently displays a stronger total reducing ability than Vc at equivalent mass concentrations. This observation indicates that the mangiferin extract derived from mango peel possesses potent total reducing capabilities and demonstrates a notable dose-dependent relationship, which can provide protection against oxidative damage and help delay cellular aging.

The antioxidant experiment results show that mangiferin has a strong scavenging ability on DPPH·, ABTS^+^·, and O_2_^−^·. At concentrations above 0.2 mg/mL, the scavenging rate of all three free radicals is more than 90%, and the scavenging rate of DPPH· is higher than Vc, indicating that the mangiferin from mango peel has a good antioxidant capacity.

### 3.6. Antidiabetic Assay (In Vitro)

#### 3.6.1. Determination of the Inhibitory Ability of Mangiferin Extracts on α-Amylase Enzyme Activity

**Figure 9:**
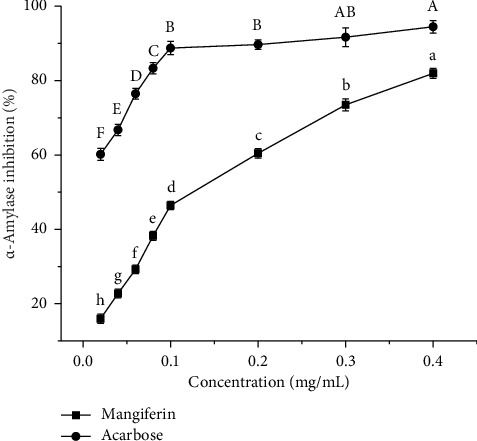
Inhibition activity of α-amylase by mangiferin and acarbose. Different letters (A–F) and (a–h) in the same line indicate significant differences (*n* = 3, *p* < 0.05).

α-Amylase is a crucial digestive enzyme that breaks down starch into reducing sugars in the digestive organs. These sugars are quickly absorbed into the bloodstream, causing blood sugar levels to rise. The therapeutic approach to treating Type 2 diabetes mellitus (T2DM) is to delay absorption of glucose through inhibition of α-amylase activity [39, 40]. As can be seen from Figure 9, the inhibitory activity of α-amylase increases gradually with higher mass concentrations of mangiferin extract and acarbose. At equivalent mass concentrations, the inhibitory activity of α-amylase^·^ by mangiferin extract is lower than that of acarbose. For mangiferin extract concentrations between 0.02 and 0.4 mg/mL, the inhibitory activity of α-amylase increases significantly (*p* < 0.05). At 0.4 mg/mL, the inhibitory activity of mangiferin extract on α-amylase reaches a maximum of 81.94%%, which is 86.74% of acarbose's inhibitory activity (94.47%) at the same concentration. This result was higher than the inhibitory activity of mangiferin extract on α-amylase reported by Kaur et al. [12] (the maximum was 77.6%). The IC_50_ values for inhibitory activity of α-amylase by mangiferin extract and acarbose were 0.1952 mg/mL and 0.05832 mg/mL. This indicates that mangiferin extract had a strong inhibitory effect on α-amylase activity, but its inhibitory effect was lower than that of acarbose.

#### 3.6.2. Determination of the Inhibitory Ability of Mangiferin Extracts on α-Glucosidase Enzyme Activity

**Figure 10:**
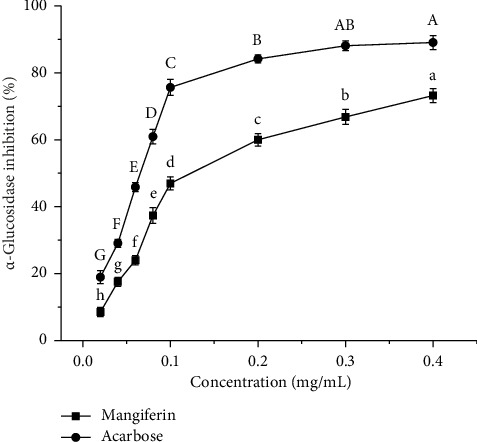
Inhibition activity of α-glucosidase by mangiferin and acarbose. Different letters (A–G) and (a–h) in the same line indicate significant differences (*n* = 3, *p* < 0.05).

α-glucosidase is located in the brush border of the small intestine and breaks down maltose and maltotriose into glucose [33]. Together with α-amylase, these enzymes enhance carbohydrate digestion and absorption, which leads to elevated blood sugar levels after meals. Previous studies have found that the blood glucose levels of patients with T2DM can be effectively controlled by regulating the activity of these two enzymes [41]. Mangiferin acts as a noncompetitive inhibitor, inhibiting α-amylase and α-glucosidase activity and reducing starch degradation, thereby controlling blood glucose peaks [42]. As can be seen from Figure 10, the inhibitory activity of α-glucosidase increases significantly (*p* < 0.05) with higher mass concentrations of mangiferin extract. For mangiferin extract concentrations between 0.02 and 0.4 mg/mL, the inhibitory activity of α-glucosidase^·^ by mangiferin extract is lower than that of acarbose. At 0.4 mg/mL, the inhibitory activity of mangiferin extract on α-glucosidase reaches a maximum of 73.19%, which is 89.04% of acarbose's inhibitory activity (82.19%) at the same concentration. The IC_50_ values for inhibitory activity of α-glucosidase by mangiferin extract were 0.08696 mg/mL, higher than acarbose's IC_50_ value of 0.06791 mg/mL. This indicates that the mangiferin extract from mango peel demonstrates relatively better inhibitory effects on α-glucosidase. These results are consistent with other authors [43]. Mangiferin extracts can be considered as a natural anti-α-amylase and α-glucosidase preparation and can also be used as a functional ingredient to control T2DM.

## 4. Conclusions

This study optimized the process of extracting mangiferin from mango peel by the ultrasound-assisted enzymatic method (enzyme content 0.10%, enzymatic hydrolysis for 44 min, ethanol 70%, ultrasonic temperature 66°C, power 300 W, and time 45 min), and the extraction rate reached 3.63 ± 0.04%, which were 3.90 times and 1.78 times that of the single-enzyme method and the ultrasonic method, respectively. It indicates that this method is efficient and time-saving. In vitro antioxidant experiments have shown that mangiferin extract from mango peel has strong antioxidant properties (DPPH^·^, ABTS^+·^, and O_2_^−·^, EC_50_ values are 0.03829, 0.06032, and 0.04949 mg/mL, respectively), and its total reducing power is superior to that of V_C_. It can effectively inhibit α-amylase (IC_50_: 0.1952 mg/mL) and α-glucosidase (IC_50_: 0.08696 mg/mL) and is a potential natural antioxidant, providing a basis for the high-value utilization of mango processing waste. However, this study also has some limitations. First, the applicability of the proposed approach to other plant sources remains unvalidated. Second, the optimized parameters are confined to laboratory scale, and their industrial feasibility requires further verification. Third, mangiferin was not subjected to purification, resulting in insufficient exploration of its functional properties. Accordingly, future research could focus on the following directions: expanding the range of plant sources and optimizing analytical methods; developing continuous flow reaction devices and optimizing conditions to facilitate industrial scaling-up; and emphasizing mangiferin purification, along with investigations into its antioxidant mechanisms, pharmacodynamics, safety, bioavailability, and potential new applications.

## Figures and Tables

**Table 1:** Factors and levels of response surface experiments.

Levels	Factor			
	Enzymolysis time (A) (min)	Enzyme addition amount (B) (%)	Ethanol concentration (C) (%)	Ultrasonic temperature (D) (°C)
−1	20	0.08	65	55
0	40	0.1	70	65
1	60	0.12	75	75

**Table 2:** Results of the negative control test.

Sample no.	Quality of mangiferin standard (g)	Actual quantity (g)	Average content (g)	RSD (%)
1	1.0002	0.99	0.99 ± 0.01	0.8
2	1.0002	1.00		
3	1.0003	0.99		

**Table 3:** Results of the precision test.

No.	1	2	3	4	5	6
Extraction rate (%)	3.20	3.19	3.22	3.22	3.18	3.20
RSD (%)	0.50					

**Table 4:** Results of the repeatability test.

No.	1	2	3	4	5	6
Extraction rate (%)	3.48	3.48	3.51	3.53	3.50	3.51
Average extraction rate (%)	3.50 ± 0.02					
RSD (%)	0.55					

**Table 5:** Results of the stability test.

No.	1	2	3	4	5	6
Extraction rate (%)	3.50	3.49	3.48	3.50	3.49	3.49
RSD (%)	0.24					

**Table 6:** Results of the recovery test.

Sample no.	Sample quality (g)	Mangiferin content (mg)	Amount added (mg)	Actual quantity (mg)	Recovery rate (%)	Average recovery rate (%)	RSD (%)
1	1.0000	34.29	2.1	36.31	96.19	99.52 ± 1.97	1.98
2	1.0001	34.91	2.1	37.00	99.52		
3	1.0003	35.52	2.1	37.55	101.43		
4	1.0002	35.06	3.1	38.21	101.61		
5	1.0003	35.68	3.1	38.75	99.03		
6	1.0002	34.90	3.1	37.98	99.35		

**Table 7:** Box–Behnken of central composite experiment design and response values.

No.	Variable				Extraction rate (%)
	A (min)	B (%)	C (%)	D (°C)	
1	40	0.10	70	65	3.76 ± 0.02
2	40	0.10	70	65	3.61 ± 0.02
3	40	0.10	65	75	3.24 ± 0.04
4	40	0.12	75	65	3.25 ± 0.03
5	60	0.10	75	65	3.34 ± 0.04
6	60	0.12	70	65	3.43 ± 0.04
7	40	0.10	75	55	3.35 ± 0.03
8	20	0.12	70	65	3.15 ± 0.01
9	20	0.08	70	65	3.28 ± 0.03
10	40	0.08	65	65	3.08 ± 0.02
11	40	0.10	70	65	3.64 ± 0.02
12	20	0.10	75	65	3.21 ± 0.02
13	40	0.10	70	65	3.71 ± 0.04
14	40	0.12	70	75	3.29 ± 0.05
15	40	0.12	65	65	3.11 ± 0.06
16	60	0.08	70	65	3.32 ± 0.05
17	60	0.10	70	55	3.35 ± 0.03
18	20	0.08	70	55	3.19 ± 0.05
19	20	0.10	65	65	2.95 ± 0.03
20	40	0.10	70	65	3.72 ± 0.02
21	60	0.10	65	65	3.23 ± 0.02
22	20	0.10	70	75	3.24 ± 0.01
23	60	0.10	70	75	3.35 ± 0.04
24	40	0.08	70	75	3.42 ± 0.03
25	20	0.10	70	55	3.17 ± 0.06
26	40	0.12	70	55	3.12 ± 0.04
27	40	0.10	65	55	2.89 ± 0.02
28	40	0.10	75	75	3.23 ± 0.02
29	40	0.08	75	65	3.31 ± 0.03

**Table 8:** Analysis of variance for the response surface quadratic model.

Source of variance	Sum of squares	df	Mean square	*F* value	*p* value	Significance
Model	1.26	14	0.09	32.88	< 0.0001	^∗∗∗^
A	0.087	1	0.087	31.78	< 0.0001	^∗∗∗^
B	5.21 × 10^−3^	1	5.21 × 10^−3^	1.91	0.1887	
C	0.12	1	0.12	43.26	< 0.0001	^∗∗∗^
D	0.041	1	0.041	14.97	0.0017	^∗∗^
AB	0.014	1	0.014	5.28	0.0375	^∗^
AC	5.63 × 10^−3^	1	5.63 × 10^−3^	2.06	0.1730	
AD	1.23 × 10^−3^	1	1.23 × 10^−3^	0.45	0.5137	
BC	2.03 × 10^−3^	1	2.03 × 10^−3^	0.74	0.4034	
BD	9.00 × 10^−4^	1	9.00 × 10^−4^	0.33	0.5748	
CD	0.055	1	0.055	20.25	0.0005	^∗∗∗^
A^2^	0.25	1	0.25	91.04	< 0.0001	^∗∗∗^
B^2^	0.27	1	0.27	99.37	< 0.0001	^∗∗∗^
C^2^	0.58	1	0.58	213.19	< 0.0001	^∗∗∗^
D^2^	0.31	1	0.31	113.18	< 0.0001	^∗∗∗^
Residual	0.038	14	2.73 × 10^−3^			
Lack of fit	0.023	10	2.31 × 10^−3^	0.61	0.7587	
Pure error	0.015	4	3.77 × 10^−3^			
Sum	1.29	28				

*Note:p* < 0.05 indicates that the model or the investigated factor has a significant influence; *p* < 0.01 indicates that the model or the investigated factor has a highly significant influence; *p* < 0.001 indicates that the model or the investigated factor has an extremely significant influence. *R*^2^ = 0.9705; *R*_Adj_^2^ = 0.9410; *R*_Pred_^2^ = 0.8789.

^∗^significant.

^∗∗^highly significant.

^∗∗∗^extremely significant.

## Data Availability

All data generated or analyzed during this study are included in this published article.
